# DNA or Protein Methylation-Dependent Regulation of Activator Protein-1 Function

**DOI:** 10.3390/cells10020461

**Published:** 2021-02-21

**Authors:** Eunji Kim, Akash Ahuja, Mi-Yeon Kim, Jae Youl Cho

**Affiliations:** 1 Department of Integrative Biotechnology and Biomedical Institute for Convergence at SKKU (BICS), Sungkyunkwan University, Suwon 16419, Korea; im144069@gmail.com (E.K.); akashahuja1988@gmail.com (A.A.); 2 School of Systems Biomedical Science, Soongsil University, Seoul 06978, Korea

**Keywords:** epigenetic, cell signaling, DNA methylation, histone methylation, protein methylation, methyltransferase, activator protein 1 (AP-1)

## Abstract

Epigenetic regulation and modification govern the transcriptional mechanisms that promote disease initiation and progression, but can also control the oncogenic processes, cell signaling networks, immunogenicity, and immune cells involved in anti-inflammatory and anti-tumor responses. The study of epigenetic mechanisms could have important implications for the development of potential anti-inflammatory treatments and anti-cancer immunotherapies. In this review, we have described the key role of epigenetic progression: DNA methylation, histone methylation or modification, and protein methylation, with an emphasis on the activator protein-1 (AP-1) signaling pathway. Transcription factor AP-1 regulates multiple genes and is involved in diverse cellular processes, including survival, differentiation, apoptosis, and development. Here, the AP-1 regulatory mechanism by DNA, histone, or protein methylation was also reviewed. Various methyltransferases activate or suppress AP-1 activities in diverse ways. We summarize the current studies on epigenetic alterations, which regulate AP-1 signaling during inflammation, cancer, and autoimmune diseases, and discuss the epigenetic mechanisms involved in the regulation of AP-1 signaling.

## 1. Introduction

Activator protein 1 (AP-1) comprises various transcription factor complexes involved in various cellular and physiological responses. It is recognized as a prime combination of extracellular signals, by which cells adapt to changes in their environment [1,2]. Activation of AP-1 has been linked to the cause of various severe diseases, which include fibrosis, organ injury, and various inflammatory disorders like rheumatoid arthritis, asthma, and psoriasis by increasing transcription of inflammatory and cellular damaging genes such as cytokines, chemokines, inflammatory mediators, and matrix metalloproteinases [3,4,5,6,7]. In addition, AP-1 activation also results in cancer progression, which is often dysregulated and contributes to tumor progression, disease aggressiveness, and resistance to drug treatment by transcriptional elevation of oncogenic proteins involved in the regulation of cell-cycle, apoptosis, survival, migration, infiltration, invasion, and proliferation of cells (Table 1) [8,9,10,11]. In contrast, AP-1 components can act as tumor suppressors that affect upstream oncogenic events such as the MAPK pathway activity [12]. The disruption of AP-1 can trigger different constituents, which stabilize the functional activation of AP-1 proteins. To curtail the role of AP-1 in various pathologies, targeting AP-1 has been proven to be an attractive therapeutic strategy [13].

The regulation of AP-1 activity results in post-translational modifications (PTMs) like the phosphorylation of Tyr, Ser, or Thr and the methylation of Lys or Arg residues [18]. In comparison to protein phosphorylation, methylation is a relatively new area of research. The knowledge of AP-1 regulation through methylation is still incomplete. Thus, in this review, we summarize the putative findings on how methyltransferases and protein substrates interact and regulate AP-1 signal transduction differentially, with a focus on how these epigenetic patterns influence the severity and heterogenicity of diseases. Moreover, we explore how we can implement these mechanisms in the development of therapeutics.

Activator protein 1 (AP-1) protein dimers consist of multigene families of proteins and transcriptional factors consisting of the Fos proteins (c-Fos, v-Fos, Fos B, Fra-1, and Fra-2); Jun proteins (c-Jun, v- Jun, Jun B, and Jun D); activating transcription factors (ATF1-4, ATF6, B-ATF, and ATFx); and musculoaponeurotic fibrosarcoma (Maf) proteins (c-Maf, Maf B, Maf G/F/K, and Nrl) [19,20,21,22]. These proteins contain conserved basic region leucine zippers (bZIPs) that are responsible for AP-1 dimerization and DNA binding. AP-1 subunits form homodimers or heterodimers for activation and recognize the consensus AP-1 sites TGAG/CTCA, also known as phorbol 12-*O*-tetradecanoate-13-acetate (TPA), or TPA-responsive elements [21]. AP-1 is an important transcription factor that modulates diverse cellular processes, including cell survival, differentiation, apoptosis, and development. AP-1 is activated by stimuli, including cytokines, chemokines, stress signals, hormones, and oncogenic stimuli. AP-1 activity is modulated at both the transcriptional and post-translational levels [21,23]. In post-translational regulation, the phosphorylation of AP-1 subunits activates transcriptional abilities that are preferentially mediated by mitogen-associated protein kinases (MAPKs) [23,24,25], since there are numerous reports that MAPKs or their upstream molecules control AP-1 activation [24,26,27,28]. 

**Figure 1:**
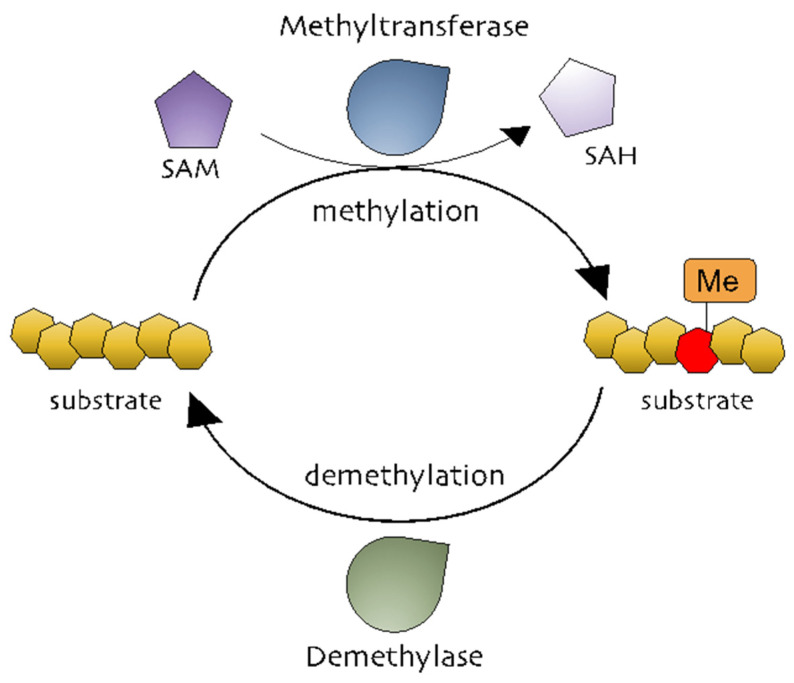
The methylation processes. Methyltransferases transfer a methyl group from a methyl donor (S-adenosylmethionine [SAM]) to a substrate. Methylated substrates can be demethylated by demethylases.

Methylation is the addition of a methyl group (-CH_3_) to a substrate and is mediated by methyltransferases to regulate biochemical responses [29]. The methyl group is donated by S-adenosylmethionine (SAM, also known as AdoMet), which is converted into S-adenosylhomocysteine (SAH, also known as AdoHcy) (Figure 1) [30,31]. Methylation occurs on DNA, RNA, and proteins (Figure 1). DNA methylation is a powerful key regulator in epigenetic gene transcription. The DNA methyltransferase (DNMT) family, including DNMT1, DNMT2, DNMT3A, DNMT3B, and DNMT3L, catalyzes a process in which the carbon atoms of the cytosine bases of cytosine-guanine pairs, often called CpG-islands (chromosomal locations rich in cytosine-guanine and p, a phosphate group between DNA bases) [32,33,34]. Of the three DNMTs, DNMT1 is associated with the epigenetic recovery of tissue [35]. Protein methylation is a post-translational modification and can occur on arginine (R), lysine (K), histidine (H), and carboxyl groups [36,37]. Members of the histone family, including H2A, H2B, H3, and H4, are well-known methylated proteins and are generally methylated on lysine and arginine residues. Methylated histones can change chromatin structure, thereby modulating gene expression [29,36]. Non-histone methylated proteins have also been reported to regulate cellular processes. Protein lysine methyltransferases (PKMTs) and protein arginine methyltransferases (PRMTs) are representative methyltransferase families. PKMTs generate three types of methylated lysine: monomethyl, dimethyl, and trimethyl lysine [38]. In comparison, three different forms of methylated arginine are generated by PRMTs: monomethyl arginine, asymmetric dimethyl arginine, and symmetric dimethyl arginine [39]. While it is well-known that AP-1 is functionally active in various physiological and pathophysiological conditions, the exact mechanisms that control the AP-1 activation pathway in terms of the methylation reaction of AP-1 and its activation pathway still remain unclear.

## 2. DNA Methylation and AP-1 Signaling

### 2.1. DNA Methylation and Cancer 

Accumulating evidence and extensive research have proven that aberrant DNA methylation to be an important epigenetic modulation in activating transcription factors and results in inflammation or different diseases [40]. Small molecule inhibitors of DNA methyltransferase (DNMT), also called as hypomethylating agents, are largely used in therapies for the treatment of myeloid splastic syndrome (MDS), myeloid leukemia. 5-Azacytidine (5-Aza), 5-aza-2’deoxycytidine (decitabine) and SGI-110 (guadecitabine) are DNMT proteins leading to DNA hypomethylation [41,42,43].

Inhibiting or blocking ERK-MAPK signaling significantly reduced DNMT1 protein and gene expression in the SW116 colon cancer cell line, ERK-MAPK inhibitor rottlerin (20 µM) resulted in p16^INK4A^ and p21^WAF1^ demethylation, which provides a direct link between ERK-MAPK signaling and DNA methylation [44]. Furthermore, a recent investigation showed ROS in the regulation of CDH-1 and E-cadherin plays an important role in the development and progression of breast cancer, by using H_2_O_2_ (40 µM) authors have shown induction of cellular migration, DNMT1, HDAC, Snail and Slug (downstream of ERK pathway) and decrease in E-cadherin gene expression and enrichment of H3K9me3 and H3K27me3 in CHD-1 promoter in MDA-MB231 and MCF-7 breast cancer cell lines, treatment of U0126 (ERK inhibitor) reduced gene expression of DNMT-1, Snail and Snug with an increase in E-cadherin and CDH-1, which implies that CDH-1 is symbiotically modulated DNA, histone methylation with histone deacetylation and results in chromatin remodeling and activation of Snail and Snug through the ERK pathway [45]. Hypermethylation of tumor suppressor genes have been related to smoking; exposure to nicotine (10 nM and 10 µM) in human pancreatic epithelial cells resulted in up-regulation of DNMT3A and 3B protein expression with activation of the acetylcholine receptor (a7nACHR) and ERK1/2, JNK and p38 MAPK, combination of ERK1/2 (U0126) and p38 (SB203580) resulted in downregulation of DNMT3A and 3B [46].

Gliomas are the most common forms of brain tumor with poor clinical results and a lower survival rate [47] Relationship between c-Jun, DNMT-1 and global methylation was studied in higher and lower grade gliomas, DNMT-1 mas, with cogene expression being 4.57-fold higher in low grade gliomas in comparison to high grade gliorrelated with overall CpG methylation levels; TCGA (tumor cancer genome atlas analysis ) analysis found that DNMT-1 was also associated with better survival in low grade gliomas with high phosphorylation of c-Jun and high CpG methylation in low grade gliomas. Patient-derived glioblastoma (BTSC168) cells treated with anisomycin increased phosphorylation of JNK, c-Jun and DNMT-1 expression levels and a significant increase in genome wide DNA methylation of promotor regions, whereas JNK inhibitor (SP600125, 50 µmol/mL) reduced protein levels of c-Jun, JNK and DNMT-1 with a reduction in global DNA methylation in CL3021 cells. The results concluded that phospho-c-Jun controls DNMT-1 expression and regulates DNA methylation in glioblastoma [48]. Studies on nasopharyngeal carcinoma (NPC) showed LMP1 induced DNMT-1 protein and RNA expression NP69 (stably expressed LMP1), in addition to siRNA targeting of JNK, c-Jun and TRADD, LMP1-YYD domain and LMP1 observed reduced DNMT-1 expression by 20%, 40%, 60% and 50%. NPC biopsy from 32 patients showed high c-Jun and DNMT-1 and LMP-1 protein expression in 27 of 32 patients (84.38%), which suggests a significant correlation between LPP1-C-Jun-DNMT1 proteins [49].

Subsequent studies have examined the increase of CD38 cell surface expression in multiple myeloma (MM) and could improve daratumumab efficacy and cell resistance. A recent study found that DNA methylation represses CD38 (CpG island in first exon) expression based on ENCODE data. MM cell lines RPMI-8226, MM.1S, XG-1 and KMS12-PE with an increasing dose of azacytidine (AZA) (1–3 µM) resulted in 1.2–2.4-fold increase in CD38 MFI in all the MM cell lines (Table 2). Furthermore ChIP-seq data resulted in transcription factor PU.1 and ATF2 involved in the regulation of CD38 expression; however, knockdown of these genes did not alter AZA-induced CD38 expression and resulted in an increase in TNF-α expression. Cotreatment of AZA with a TNF-α neutralizing antibody completely abrogated CD38 expression in MM plasma cells, suggesting that the TNF-α pathway may play an important role in orchestrating this process [50].

It is shown that CpG methylation in mammalian DNA is known to increase the binding of c-Jun/c-Fos heterodimer [51]. A recent technique involving the use of microfluidic-based ligand enrichment followed by Smile-seq showed a significant difference between DNA binding motifs Jun homodimers and heterodimers [51]. Jun-Fos heterodimers strongly binds to the TPA-response element (TRE). In another report, the analysis of CpG DNA methylation showed that c-Fos/c-Jun heterodimers bind more strongly to mCGACTCA than unmethylated CGACTCA [52].

### 2.2. DNA Methylation and Osteoporosis

Profound loss of bone leads to changes in skeletal architecture and integrity, and results in disuse osteoporosis (DOP). A study revealed that DNMT-1 levels were significantly higher in hindlimb unloading (HLU) rats with DOP with decreased expression levels of H19 after 3 and 4 weeks with inhibited ERK signaling pathways in DOP bone tissues. The in vivo knockdown of DNMT-1 using SiRNA in Sprague-Dawley (SD) rats significantly upregulated H19 expression with a decrease in CpG methylation rates of 37.0% in the siDNMT group and activation of MAPK-ERK, which prevented the development of DOP; these results suggest that targeting DNMT-1-H19-ERK signaling can be used as a new strategy for treating DOP [53]. A similar work by Lorenzo et al. examined DNA methylation patterns during osteoclastogenesis (OC) [54]. Analysis of TRANSFAC data during OC differentiation revealed the hypomethylation of transcription factors like AP-1 (39%) and NF-κB (15%) with greater enrichment in PU.1 PU.1 is critically involved in OC differentiation and the regulation of cytokine expression and is located upstream of NF-κB. The knockdown of PU.1 in monocytes using siRNA resulted in impaired DNA methylation and reduced TET2 and DNMT3b during OC differentiation.

### 2.3. DNA Methylation and Inflammation

**Figure 2:**
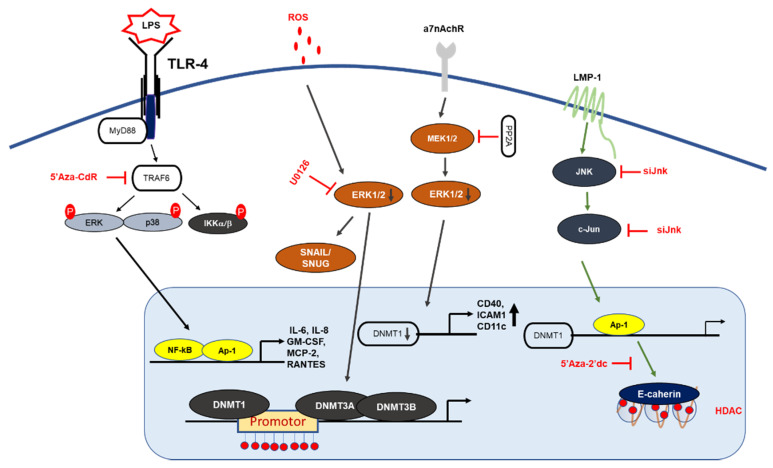
MAPK signaling mechanism by DNA methylation. LMP1 induction of DNMT1 results in hypermethylation of E-cadherin promoter: LMP1 activates the signaling of AP-1, which then binds to the DNMT-1 promoter, leading to hypermethylation of the E-cadherin gene. Deletion of the DNMT1 component increases LPS-induced cytokine expression in NF-κB and AP-1 signaling. PP2A dephosphorylates MEK1/2 and inhibits the activity of DNMT1, leading to the hypomethylation and increased expression of CD70 and CD11a. In T-cells, 5-Aza-Cdr treatment significantly increased the gene expression levels of cytokine IL-6, IL-8 GM-CSF, MCP2, and RANTES, and resulted in the upregulation of ERK phosphorylation.

DNA methylation is also known to regulate TLRs, TNF-receptor associated factor 6 (TRAF6), and myeloid differentiation primary response 88 (MyD88) adaptor proteins, which suggests that DNA methylation can epigenetically regulate inflammatory signaling [44]. A recent report on dental pulp inflammation in human dental pulp cells (hDPCs) challenged with lipopolysaccharide (LPS) decreased DNMT1, IL-6, and IL-8 mRNA gene expression within 24 hours; furthermore, the knockdown of DNMT1 by siRNA increased IL-6 and IL-8 gene expression with activation of the NF-κB and MAPK signaling pathways, suggesting that DNA methylation plays an important role in inflammatory responses [55] (Figure 2). Similar studies on dental caries, a chronic, infection, and destructive disease, observed a decrease in DNMT-1 with a decrease in the gene expression levels of inflammatory cytokines, whereas the knockdown of DNMT-1 resulted in increases in p38 and ERK in the MAPK pathway and resulted in the hypermethylation of the MyD88 adaptor protein in lipoteichoic acid (LTA)-stimulated human odontoblast-like cells (hoBs) [56]. The above studies demonstrate that DNMT-1 is an important regulator of epigenetic modification in controlling inflammation.

Human immunodeficiency virus type-1 (HIV-1) infection results in changes in gene expression patterns and the induction of transcription factors NF-κB and AP-1, which regulate DNMT-1 expression. A study on HIV-1 by Youngblood and Reich [57] showed that DNMT-1 luciferase activity was increased by 6- to 7-fold in co-transfected HIV-1 cDNA; blocking AP-1 activity using resveratrol (15 µM) reduced the HIV-1 induction of pGL3^DNMT1-SV40^ (hybrid construct), so this study suggested that the inhibition of DNMT-1 could reduce HIV-1 pathogenesis [57].

### 2.4. DNA Methylation and Autoimmune Disease 

Systemic lupus erythematous (SLE) is an autoimmune disorder in females characterized by the production of autoantibodies and results in epigenetic changes in DNA and histone [58]. Procainamide, a DNMT inhibitor inhibits ERK pathways by downregulating DNMT expression in lupus T cells [59] (Table 2). Hydralazine suppressed the upregulation of DNMT1 and DNMT3a activity through the Ras/MAPK signaling pathway without inhibiting DNMT activity. Inhibiting ERK and DNA methylation using an inhibitor decreased DNA methyltransferase in lupus T cells, suggesting that DNA methylation and ERK inhibitors may be relevant in lupus recovery and may contribute to the development of autoimmunity [60]. A recent study showed that the silencing of protein phosphatase 2A (PP2Ac) in T-cells using siRNA resulted in increased DNMT1 expression and MEK/ERK phosphorylation with reduced expression of CD70 and ITGAL (methylation-sensitive genes). T-cells isolated from SLE patients also resulted in similar patterns; these reports suggest a potential link between pp2Ac and DNMT1 [61]. A lupus-inducing drug, hydralazine, is reported to contribute to lupus disease pathogenesis [62]. Hydralazine inhibited the ERK pathway, which resulted in the hypomethylation of DNMT1 and DNMT3a in T cells [62]. The results suggest that the inhibition of MAPK/ERK signaling is important for DNA methylation in T cells. 

## 3. Histone Methylation and AP-1 Signaling

### 3.1. Histone Methylation and Cancer 

**Figure 3:**
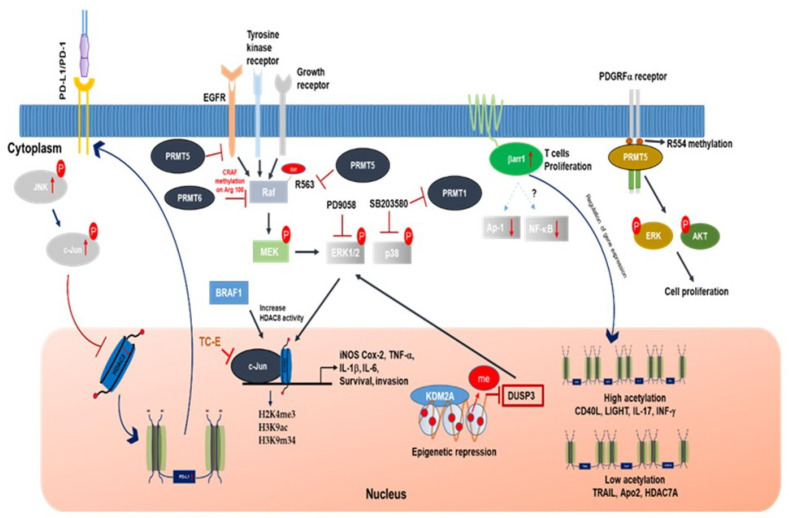
Epigenetic regulation of AP-1 subunits through histone methylation in T cells. In drug-resistant cancer cells, increased JNK phosphorylates c-Jun and translocates it into the nucleus, which inhibits HDAC3 expression and induces the H3 acetylation of the PD-L1 promoter, which increases PD-L1 expression. In addition, βarr1 increased T cell proliferation, downregulated NF-κB and AP-1 signaling and promoted the acetylation of histone H4 in the CD40L, LIGHT, IL-17, and INF-γ promoter regions while downregulating the histone acetylation of the TRAIL, Apo2, and HDAC7A promoter regions.

The role of TCF-1 and AP-1 interaction was studied in coronary artery disease (CAD). AP-1 activation binds to SMAD3 and CDKN2BAS, engaging H3K27 acetylation activity and resulting in histone modification and open chromatin, which in turn promotes TCF-1 binding recruits HDAC1 and HDAC2 to SMAD3 and CDKN2BAS loci to deacetylate H3K27 and results in the suppression of transcription. AP-1 and TCF-21 are linked to CAD, which represent a unique mechanism in human disease [63]. The combination of HDAC inhibitor (NaB, 1.0 or 2.5) and MAPK/ERK (U0126,12.5 or 25 µM) decreased CD133 and BMI1 gene expression in Daoy and D283 cell lines, inhibited medulloblastoma (MB) neurosphere formation and reduced MB proliferation [64]. Furthermore, the combination of the BRAF and MEK inhibitor results in an increase in HDAC8 expression in melanoma cells and this increase leads to the regulation of MAPK and AP-1 signaling cascades through EGFR and proto-oncogene MET, which contributes resistance to the BRAF and MEK inhibitors and melanoma cells expressing HDAC8 observed resistance to the BRAF inhibitor treatment with nuclear translocation of c-Jun. Whereas, HDAC8 knockdown inhibited BRAF resistance and decreased tumor size (Figure 3). The combination of small molecule HDAC inhibitor Panobinostat and PCI-30451 significantly reduced tumor burden and enhances the sensitivity to BRAF inhibitors proving HDAC8 inhibitors as a promising role in therapeutics [65]. Targeting signaling pathways by using small inhibitors or HDACs inhibitors may overcome resistance to BRAF inhibitors; therefore, combination treatment has proven to be effective as an anti-cancer therapy. A combination of BRAF (dabrafenib) and MEK (trametinib) inhibitors is used for the treatment of metastatic melanoma with BRAF^V600E^ mutation and these inhibitors observed an increase in KIT expression (tumor suppressor gene) and induced alterations in CCND1, RB1, and MET in patients with metastatic melanoma [66]. Chemotherapy is one of the most popular reliable strategies to treat cancer, even though there is a positive response of chemotherapy, and most patients develop drug resistance [67]. A recent study observed that the PD-L1 gene and protein expression increased in drug resistance cell lines, A549, MCF-7 and HepG2, and this increase was significantly inhibited by c-Jun knockdown, suggesting that JNK/c-Jun signaling is activated in drug resistance cell lines and increases PD-L1 expression, which in turn decreases HDAC3 levels [68]. Histone modification of arginine or lysine residue regulates gene expression and cell signaling pathways [69]. A study on NSCLC tumor and the lung cancer cell line found that histone H3 lysine 36 (H3K36) demethylase KDM2A (FBXL11 and JHDM1A) activates ERK1/2 by the epigenetic regulation of DUSP3 [70]. Furthermore, overexpression of KDM2A in low KDM2A NSCLC cell line increased cell proliferation and invasion capabilities, and increased ERK1/2 through a decrease in the dual-specificity phosphatase-3 (DUSP3) gene by demethylating H3K36me2 at the DUSP2 promotor [70]. Consistently knockdown of KDM2A significantly decreased tumor growth and invasive capabilities in mouse xenograft models.

Increase in histone deacetylation activity is usually observed in hepatocellular carcinoma (HCC) patients [71]. A HDAC inhibitor quisinostat (JNJ-26481585) treatment significantly induced G0/G1 cell cycle arrest in the HCC cell line with a dose dependent increase in phosphorylation of JNK and c-Jun; furthermore, combination treatment using quisinostat and sorafenib markedly reduced JNK phosphorylation and induced apoptosis, suggesting that combination therapy could be useful in recovering patients from the burden of hepatocellular carcinoma [72].

### 3.2. Histone Methylation and Inflammation 

Reduced levels of the sigma-1 receptor (Sig1R) leads to neuroinflammatory diseases like Alzheimer’s. A recent study found that the administration of LPS resulted in a decrease in SigR1 mRNA levels in a concentration-dependent manner in rat primary cultured microglia. Co-treatment with transforming growth factor beta-activated kinase 1 (TAK-1) (TAK, 0.3 µM), p38 MAPK (SB239063, 3 µM), and HDAC6 (tubastatin A, 1 µM) inhibitors significantly restored Sig1R expression in LPS-treated microglia [73]. However, work related to HDAC2 has been documented to attenuate LPS-induced inflammatory signaling by regulating c-Jun and PAI-1 expression in RAW264.7 macrophages [74]. Theophylline (TM5275) (0, 10, and 20 µM), an HDAC2 activator, reduced the mRNA gene expression of inflammatory cytokines PAI-1, TNF, and MIP-2 in LPS-stimulated RAW264.7 cells; the knockdown of HDAC2 using siRNA promoted the binding of NF-κB p65 and c-Jun to the PAI-1 gene promoter region, while the inhibition of PAI-1 alone or in combination (10 µM) with theophylline (100 µM) significantly inhibited the gene expression of PAI-1, TNF, and MIP-2 mRNA expression levels, which suggests that HDAC2 can reduce LPS-induced inflammatory signaling.

The regulation of HDAC expression can epigenetically control an array of genes involved in inflammation and pain [75]. The levels of HDAC1 were increased in spinal dorsal horn seven days after surgery [76]. Spread nerve injury (SNI) mice displayed high protein expression levels of HDAC1, JNK, and c-Jun at 1 and 3 weeks in the ipsilateral side (ipsi), but treatment with the HDAC1 inhibitor LG325 (5 µg per mouse) prevented c-Jun stimulation. A double immunostaining experiment confirmed the localization of p-JNK, HDAC1, and c-Jun in the astrocytes of SNI mice. Short chain fatty acids (VPA, 2 µM), hydroxyamic acids (TSA, 500 nM and SAHA, 1 µM) and synthetic benzamide derivatives (M344, 1 µM), all inhibitors of HDAC, transcriptionally decreased c-Jun/MEK1/2-ERK1/2, FRA1, and Raf decreased the cell viability of neuroblastoma cell lines SH-SY5Y and SK-N-SH; these results suggest the potential use of HDAC inhibitors as therapeutics [77]. A recent study on HDAC2 revealed that HDA2 directly interacts with a c-Jun promoter by inhibiting a series of inflammatory genes, resulting in the indirect enhancement of proinflammatory genes [78].

Vascular smooth muscle cells (VSMCs) are subject to cardiovascular disease (CVD), which includes atherosclerosis and vascular remodeling after injury. The study on VSMCs in atherosclerosis models observed a decrease in the expression of H3K9me2 with increased inflammation. Furthermore, genome-wide mapping observed the enrichment of H3K9me2 levels in the inflammatory gene promoters MMP3, MMP9, MMP12, and ILThe inhibition of H3K9me2 levels by G9A/GLP increased the binding of AP-1 and NF-κB at the MPP9 and IL6 promoters; these results consolidate the critical role of H3K9me2 in the VSMC inflammatory response [79]. In another study, Oliveira et al. [80] reported that H2O2 induces Cxcl8 expression in the late phase through JNK/c-Jun/AP-1 signaling and promotes the modulation of histones H2K4me3, H3K9ac, and H3K9m34 at the Cxcl8 promoter, suggesting that H2O2 is a potential candidate for the development of anti-inflammatory treatments.

H3 phosphorylation and acetylation appear to be important in relation to drug-related behaviors. Exposure to drugs results in changes in the gene expression and methylation of H3KA study in mice showed that cocaine administration results in up-regulation of Suv39H1, an H3K9 methyltransferase. This increase resulted in the H3K9 gene promoter activity of c-Fos, which seems to be highly associated with acute cocaine use and potentiated by HDAC [81]. A recent study focused on transcriptome profiles of developmental origins and genomic and micro-anatomic relationships among phenotypically distinct macrophages in diet-induced non-alcoholic steatohepatitis (NASH) mouse models [82]. The NASH diet induced changes in Kupffer cells, with open chromatin exhibiting increased H3K27ac and the enrichment of de novo motifs matching AP-1, NFAT, RUNX, and EGR with increased mRNA expression of Atf, Fos, Jun, and Egr2 [82].

UTX/KDM6A, also known as H3K27me, is required for normal development and differentiation [83]; mutations in UTX/KDM6A usually develop into Kabuki syndrome and group 4 pediatric medulloblastoma in humans [84]. A recent study characterized the activity of UTX during neuronal differentiation. UTX binding promotes the suppression of genes involved in extracellular matrix organization, cell proliferation, and signaling pathways; in contrast, UTX-KO cells decreased neuronal differentiation and increased glial/astrocytic differentiation. Jun, JunB, FosL1, and FosL2 were upregulated in UTX-KO, in comparison to UTX-WT during neuronal development, which suggest that AP-1 transcription factors were involved in the regulation of gene expression in human pluripotent stem cells (hNSc) [85].

EZH2 is also involved in AP-1 signaling. We demonstrated a correlation between EZH2 and AP-1 proteins. The regulation of Fra-2 activity is necessary for epidermal barrier differentiation. Fra-2 regulates epidermal differentiation complex gene expression through methylation and phosphorylation [19]. In this process, Fra-2 transcriptional activities are suppressed by the transcriptional repressor EZH2, which co-occupies the binding promoter sequences of Fra-2, and then H3K27me3 is methylated. The Fra-2-mediated initiation of genes is blocked due to repressive methylation markers. Thus, gene expression is co-regulated through the occupation of an identical promoter by Fra-2 and EZH2.

### 3.3. Histone Methylation and Autoimmune Disease 

β-arrestins (βarr) are essential signaling molecules for T cell survival (Figure 3). The βarr1 expression levels were enhanced in T lymphocytes from patients with primary biliary cirrhosis (PBC), which correlated with enhanced disease activity. Furthermore, the overexpression of βarr1 resulted in T cell proliferation, increased INF-γ levels, and downregulated NF-κB and AP-1 activity, promoting the acetylation of histone H4 in CD40L, IL-17, and INF-γ promoter regions and downregulating acetylation in the promoter regions of TRAIL, Apo2, and HDAC7A, resulting in T cell gene regulation [86].

Epigenetic modifications and regulation of gene expression were studied in hippocampus-dependent, long-term memory formation in C57BL/6 mice [87]. The study found that long noncoding RNA NEAT-1 increased in the hippocampi of 24-month-old mice, which induced H3K9me2 methylation and decreased c-Fos levels. The inhibition of NEAT-1 in 24-month-old mice repressed H3K9me2 levels at the c-Fos promoter region and resulted in improvement in a memory-associated behavioral test. 

## 4. Protein Methylation

Post translational modification of proteins is a vital process that is subjected to epigenetic modification and maintains cellular machinery like transcription, translation, and cellular signaling. The activation or phosphorylation of protein kinases are known substrates of methylation. Like protein phosphorylation, protein methylation also plays a key role in the regulation of cell signaling pathways, cell proliferation, and cell differentiation. Apart from transcription factors, membrane receptors are also subjected to methylation and demethylation, which suggests that both the phosphorylation and the methylation of proteins and receptors work together in the PTM process.

### 4.1. PRMT1 and Cancer/Inflammation

AP-1 regulation by c-Jun and c-Fos results in abnormal cell proliferation and the activation of various oncogenic and carcinogenic units [88]. In addition, c-Jun activation orchestrates and regulates various coactivators or repressors of genes linked to oncogenic and carcinogenic responses [88]. A recent study identified a coactivator of c-Jun named ROCO-1 that is linked to the oncogenic activation of the AP-1 gene [89]. The molecular regulation of ROCO-1 has been identified as arginine methylation, which enhances c-Jun binding; PRMT1 specifically methylates ROCO-1 on the R98 and R109 arginine residues, which enables the binding of ROCO-1 and c-Jun. Furthermore, the depletion of PRMT1 by ShRNA or suppression of this enzyme by SB203580 inhibited ROCO-1 methylation and c-Jun binding, resulting in a decrease in gene expression (Figure 3). The above results and findings suggest that PRMT1, ROCO-1, and c-Jun control the transcription of genes and modulate cell signaling pathways [89].

Calcium ion (Ca^2+^) has been shown to mediate various cellular responses through PTM [90]. The hypothesis is that Ca^2+^ selectively regulates signaling pathways due to its wide range of binding proteins. Liu et al. were able to show the regulatory mechanism that demonstrates how PRMT1 modulates the MAPK pathway in response to Ca^2+^ [91]. Ca^2+^ stimulation significantly enhanced protein methyltransferase PRMT1 activity in cells and enhanced Arac-induced erythroid differentiation (80% at 96 h), whereas a pharmacological activator of p38 MAPKs, SB203580, completely diminished Arac-induced erythroid differentiation, indicating that the MAPK (p38) signaling pathway plays a crucial role in Ca^2+^-mediated signaling.

A recent study from our group reported that the PRMT1-selective inhibitor TC-E 5003 modulates the LPS-induced AP-1 and NF-κB signaling pathways, suggesting TC-E 5003 as a potential anti-inflammatory compound [92]. TC-E 5003 (1 µg) downregulated LPS-induced pro-inflammatory gene expression levels (COX-2, TNF-α, IL-1β, and IL-6) with decreased nuclear translocation of AP-1 (c-Jun) and NF-κB (p50 and p65) subunits; TC-E 5003 treatment also resulted in a decrease in the upstream proteins involved in AP-1 and NF-κB signaling, which further supports TC-E 5003 as a potential anti-inflammatory compound [92].

### 4.2. PRMT5 and Cancer 

**Figure 4:**
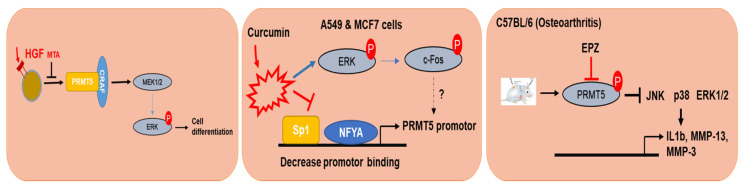
Mechanism and action of PRMT5 in different models. PRMT5-dependent methylation activates and enhances the degradation of CRAF resulting in reduced catalytic activity, and activation of downstream kinases, such as MEK1/2 and ERK1/2 in mouse melanoma cell lines. Transcription of PRMT5 was decreased by curcumin through modulation of the Akt-mTOR pathway and PKC-ERK1/2-p38-c-Fos kinase pathways.

EGFR and RAF is located upstream of MAPKs, which is regulated by PRMT5 [93] (Figure 3). In this set of experiments, PRMT5 monomethylates, the EGFR receptor at the R117 methylation site was observed and upregulates the autophosphorylation activity of EGFR at Tyr (Y) 1173, which resulted in the binding of the SH2 domain of SHPThis binding of SHP1 downregulates EGFR-ERK signaling; furthermore, the blocking of the R117 methylation site increased proliferation, migration, and invasion in breast epithelial cells [93]. The methylation of Ser/Tyr RAF1 is also regulated by PRMT5 in melanoma cells, where PRMT5 methylates RAF1 at R563, and downregulates MEK1/2-ERk1/2 protein kinases. The pharmacological inhibition of PRMT5 by 5′-methylthioadenisine (MTA) or the inhibition of PRMT5 using ShRNA increased RAS-ERK1/2 activity in response to hepatocyte growth factor (HGF) and resulted in tumor suppressive effects by downregulating EGFR and RAF1 signaling in PC12 tumor cells [94] (Figure 4).

PRMT5 regulates the PETN-Akt axis in glioblastoma cell lines (GBM) [95]. Banasavadi et al. have shown that PRMT5 knockdown resulted in apoptosis and led to G1 cycle arrest via the upregulation of pChip immunoprecipitation-PCR PRMT5 regulated and controlled Akt and ERK activity in GBM neutrospheres and GBM differentiated cells. Furthermore, the in vivo deletion of PRMT5 decreased intracranial tumor size in mice [95]. These findings suggest the significant role of PRMT5 in controlling tumors. Increased PRMT5 expression could epigenetically modify histone (H3R8me2s and H4R3me2s) methylation and promote the transcription of gene expression, resulting in cancer progression [96]. In this regard, understanding the mechanism and action of PRMT5 could lead to new therapeutics. A recent study described curcumin as a potential anti-cancer property. Curcumin (2 and 20 µM) downregulated PRMT5 and MEP50 in A549 and MCF-7 cell lines, with increases in ERK1/2 and p38 protein expression. The cotreatment of curcumin with FR180204 (0.5 µM; an ERK1/2 inhibitor) and SB203580 (100 µM; a p38 inhibitor) decreased PRMT5, NF-Y subunit A (NF-YA), and Sp1 protein expression [97] (Table 3).

Furthermore, PRMT5 activity is required for PDGFRα to carry out downstream signaling (Figure 3) [98]. The loss of PRMT5 in oligodendrocyte precursor cells (OPCs) during oligodendrocyte differentiation resulted in hypomethylation and observed post-natal death. PRMT5 deletion also resulted in the increased binding of cbl and PDGFRα at the Y555 site, which resulted in proteosomal degradation. Thus, the results suggest that the inhibition of PRMT5 could be a therapeutic target to control the PDGFRα receptor [98].

### 4.3. PRMT5 and Arthritis 

Osteoarthritis (OA) is a disease characterized by chronic joint pain that occurs at ≤ 65 years of age [99,100]. OA includes the deterioration of articular cartilage, increase in synovial inflammatory responses, development of osteophytes, and remodeling of subchondral bone [100]. A recent study reported the specific upregulation of PRMT5 in human OA chondrocytes; furthermore, the inhibition of PRMT5 by EPZ significantly inhibited cartilage destruction in DMM mice, reduced MMP3 and MMP13 expression, and also inhibited the upregulation of p65, p38, and JNK, suggesting that PRMT5 is a regulator of OA pathogenesis [101].

Lysine demethylase KDM2A activates the ERK signaling pathway by downregulating the expression of DUSP3, while PRMT5 methylates the EGFR receptor and RAF, which downregulate MAPK signaling. PRMT5 modulates PDGFRα receptor protein stability; arginine methylation at R545 of PDGFRα promotes the binding of PRMT5 and ERK as well as Akt signaling.

### 4.4. PRMT6 and Cancer 

**Figure 5:**
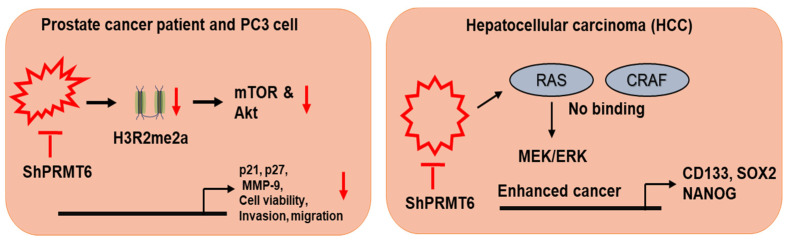
Role of PRMT5 in post translation modification. PRMT-mediated epigenetic gene activation/repression. PRMT6 knockdown in PC-3 cells displayed decrease in malignant phenotype, increasing apoptosis and decreasing cell viability, migration and invasion. PRMT6 silencing was associated with decreased H3R2me2a levels. Interaction of PRMT6 with CRAF on arginine 100, which decreased its RAS binding potential and altered its downstream MEK/ERK signaling.

The role of PRMT6 is controversial as its expression is different in different tumor types. PRMT6 can function as an activator or a repressor in different tumors [102]. Lower PRMT6 expression is observed in melanoma. However, higher expression patterns of PRMT6 are observed in bladder, lung, cervical, breast, and prostate cancers [103]. A recent study reported that silencing PRMT6 potentiated tumor metastasis, increased migration and invasion, and was resistant to cisplatin, 5-fluorouracil, and sorafenib treatment therapy in hepatocellular carcinoma (HCC) (Figure 5). Transcriptome and protein-protein analyses revealed that PRMT6 directly interacted with CRAF. Importantly, CRAF with the R100K mutation resulted in the increased phosphorylation of ERK1/2 and c-Jun (Figure 5). Furthermore, the clinical knockdown of PRMT6 in DEN+CCL4 HCC induced PRMT6 knockout mouse models to develop bigger HCC tumors in comparison to WT mice [104].

## 5. Conclusion and Perspectives

Epigenetic modification results in post translational modification and can cause uncontrolled inflammatory disorders, cancer progression, and drug resistance. During disease progression or infection, epigenetic modifiers (chromatin) alter transcription patterns (genes), which assist in the clearance of pathogens or result in evasion of the pathogens. In this regard, AP-1 transcription factors play a pivotal role in the regulation of transcription involved in various pathophysiological conditions, including inflammation and cancer. The development of epigenetic therapeutics against inflammation or tumors is based on cells of the immune system. In the above literature review, we have tried our best to address all the cell phenotypes.

DNA, histone, and protein methyltransferases have been pharmacologically studied and are known to target AP-1 signaling. Moreover, several methyltransferases and histone small molecule inhibitors have been developed and studied in various cancer cell lines, immune cells, and inflammatory diseases, but very few have played a prominent role in specifically targeting AP-1 signaling pathways through epigenetic therapies. Moreover, the combination of epigenetic inhibitors and small molecule inhibitors of MAPK/ERK is rapidly developing and becoming a novel paradigm for the treatments of cancer, autoimmune, and inflammatory disease. These combinations are coupled with next generation sequencing and technologies like machine learning, which are likely to provide guidance for rational combinations. Furthermore, transcriptome analysis at the single-cell level will greatly enhance our understanding of the epigenetic modulation of the AP-1 signaling mechanism. The ability to use and exploit these biological interactions will provide deep knowledge and opportunities for new and enhanced epigenetic therapeutics in relation to AP-1 signaling.

In addition, since studies on targeting PRMT have led to the understanding of its potential therapeutic targets in various diseases, our future goal is to further refine our knowledge and understanding of how different PRMTs regulate the AP-1 signaling mechanism in various diseases, and moreover, to accurately develop and test PRMT inhibitors in relation to cancer and immune cells, as they will result in long-term therapeutic efficacy. Finally, we described recent developments in the identification of multiple histone methyltransferases (H3R2, H2AR3, and H4R3) in various disease types. It will be very interesting to study how histone modification affects post translational modification and the activity of PRMT.

## Figures and Tables

**Table 1:** Representative genes expressed by AP-1.

Biological Function	Genes	Ref.
Inflammation	IL-1, -2, -6, -8, -15, TLR3, Cyclooxygenase 2, TNF-α, CXCL-1, -2	[14]
Migration and invasion	MMPs (1, 3, and 9), ARP2/3, autotoxin, cathepsin L, CD44, Krp-1, Ezrin, Mts-1	[15,16]
Proliferation, apoptosis, and cell cycle	TGF-α, -β, Cyclin D1, p16, FasL	[17]

**Table 2:** List of DNMT1 compounds and their roles.

Compound	Target	Disease
Azacytidine (5-Aza)	DNMT	Myeloid splastic syndrome Multiple myeloma
Decitabine (5-aza-2’-deoxycytidine)	DNMT	Myeloid splastic syndrome
Guadecitabine (SGI-110)	DNMT	Myeloid splastic syndrome

**Table 3:** Summary of compounds and inhibitors mentioned in this review.

Inhibitor	Target		Ref.
MAPK			
Rottlerin	ERK	Demethylation of p16^INK4A^ and p21^WAF1^	[44]
U0126	ERK	Reduction of DNMT-1 gene expression	[45]
U0126 and SB203580	ERK, p38	DNMT3A and DNMT3B downregulation	[46]
SP600125	JNK	Decreased protein level of c-Jun, JNK and DNMT-1	[48]
Curcumin, FR180204 and SB203580	ERK1/2, p38	Downregulation of PRMT5, NF-YA and Sp1 protein expression	[97]
Dabrafenib and trametinib	BRAF, MEK	Increased KIT expression	[66]
**Methyltransferase**			
Procainamide	DNMT	ERK pathway inhibition	[59]
Hydralazine	DNMT-1	ERK pathway inhibition	[62]
TC-E 5003	PRMT1	Downregulation of AP-1 activity	[92]
5’-methylthioadensine	PRMT5	Activation of RAS-ERK1/2 activity	[94]
Curcumin	PRMT5	Increased protein of ERK1/2 and p38	[96]
**Deacetylase**			
JNJ-26481585 (quisinostat)	HDAC	Phosphorylation of JNK and c-Jun	[77]
LG325	HDAC1	Suppression of c-Jun activation	[76]
Panobinostat, PCI-30451	HDAC	Enhancement of BRAF inhibitors	[65]
**Co-treatment**			
U0126 and NaB	ERK, HDAC	Decreased gene expression level of CD133 and BMI1	[64]
TAK, SB239063 and tubastatin	TAK, p38, HDAC6	SigR1 expression	[73]

## Data Availability

Not applicable.

## Abbreviations

5-aza-dC	5-aza-2’deoxycytidine
ADMA	asymmetric dimethyl arginine
ALA	5-aminolevulinic acid
Adox	adenosine dialdehyde
CCND1	cyclin D1
CRE	cAMP-responsive element
DNMTs	DNA methyltransferases
EDC	epidermal differentiation complex
ER	estrogen receptors
EREs	estrogen response elements
ERK	extracellular signal-regulated kinase
EZH2	enhancer of zeste homolog 2
HBV	hepatitis B virus
HBx	protein HBV X protein
HCC	hepatocellular carcinoma
HCV	hepatitis C virus
HIF	hypoxia-inducible factor
IGF-1	insulin-like growth factor-1
IL	interleukin
JNK	c-Jun N-terminal kinase
MAPKs	mitogen-activated protein kinases
MEK	MAPK/ERK kinase
MMA	monomethyl arginine
MMP	matrix metalloproteinase
MMSET	multiple myeloma SET domain
MT2A	metallothionein 2A
PDT	photodynamic therapy
PKMTs	protein lysine methyltransferases
PRMTs	protein arginine methyltransferases
PTMs	post-translational modifications
RACO-1	RING domain AP-1 coactivator-1
SAH	S-adenosylhomocysteine
SAM	S-adenosylmethionine
SDMA	symmetric dimethylarginine
SMYD3	SET- and MYND-domain containing protein 3
SRC	steroid-receptor coactivator

## References

[1] Lopez-Bergami P., Lau E., Ronai Z. (2010). Emerging roles of ATF2 and the dynamic AP1 network in cancer. Nat. Rev. Cancer.

[2] Hess J., Angel P., Schorpp-Kistner M. (2004). AP-1 subunits: Quarrel and harmony among siblings. J. Cell Sci..

[3] Zenz R., Eferl R., Scheinecker C., Redlich K., Smolen J., Schonthaler H.B., Kenner L., Tschachler E., Wagner E.F. (2007). Activator protein 1 (Fos/Jun) functions in inflammatory bone and skin disease. Arthritis Res. Ther..

[4] Gungl A., Biasin V., Wilhelm J., Olschewski A., Kwapiszewska G., Marsh L.M. (2018). Fra2 Overexpression in mice leads to non-allergic asthma development in an IL-13 dependent manner. Front. Immunol..

[5] Trop-Steinberg S., Azar Y. (2017). AP-1 expression and its clinical relevance in immune disorders and cancer. Am. J. Med. Sci..

[6] Yang W.S., Kim J.H., Jeong D., Hong Y.H., Park S.H., Yang Y., Jang Y.-J., Kim J.-H., Cho J.Y. (2020). 3-Deazaadenosine, an S-adenosylhomocysteine hydrolase inhibitor, attenuates lipopolysaccharide-induced inflammatory responses via inhibition of AP-1 and NF-κB signaling. Biochem. Pharmacol..

[7] Yang W.S., Kim H.G., Lee Y., Yoon K., Kim S., Kim J.H., Cho J.Y. (2020). Isoprenylcysteine carboxyl methyltransferase inhibitors exerts anti-inflammatory activity. Biochem. Pharmacol..

[8] Belguise K., Cherradi S., Sarr A., Boissière F., Boulle N., Simony-Lafontaine J., Choesmel-Cadamuro V., Wang X., Chalbos D. (2017). PKCθ-induced phosphorylations control the ability of Fra-1 to stimulate gene expression and cancer cell migration. Cancer Lett..

[9] Talotta F., Mega T., Bossis G., Casalino L., Basbous J., Jariel-Encontre I., Piechaczyk M., Verde P. (2010). Heterodimerization with Fra-1 cooperates with the ERK pathway to stabilize c-Jun in response to the RAS oncoprotein. Oncogene.

[10] Choi E., Kim E., Kim J.H., Yoon K., Kim S., Lee J., Cho J.Y. (2019). AKT1-targeted proapoptotic activity of compound K in human breast cancer cells. J. Ginseng Res..

[11] Ahuja A., Kim J.H., Kim J.-H., Yi Y.-S., Cho J.Y. (2018). Functional role of ginseng-derived compounds in cancer. J. Ginseng Res..

[12] Eferl R., Wagner E.F. (2003). AP-1: A double-edged sword in tumorigenesis. Nat. Rev. Cancer.

[13] Ye N., Ding Y., Wild C., Shen Q., Zhou J. (2014). Small molecule inhibitors targeting activator protein 1 (AP-1). J. Med. Chem..

[14] Wang A., Al-Kuhlani M., Johnston S.C., Ojcius D.M., Chou J., Dean D. (2013). Transcription factor complex AP-1 mediates inflammation initiated by *Chlamydia pneumoniae* infection. Cell. Microbiol..

[15] Ozanne B.W., Spence H.J., McGarry L.C., Hennigan R.F. (2006). Transcription factors control invasion: AP-1 the first among equals. Oncogene.

[16] Benbow U., Brinckerhoff C.E. (1997). The AP-1 site and MMP gene regulation: What is all the fuss about?. Matrix Biol..

[17] Shaulian E., Karin M. (2001). AP-1 in cell proliferation and survival. Oncogene.

[18] Biggar K.K., Li S.S. (2015). Non-histone protein methylation as a regulator of cellular signalling and function. Nat. Rev. Mol. Cell Biol..

[19] Wurm S., Zhang J., Guinea-Viniegra J., García F., Muñoz J., Bakiri L., Ezhkova E., Wagner E.F. (2015). Terminal epidermal differen-tiation is regulated by the interaction of Fra-2/AP-1 with Ezh2 and ERK1/2. Genes Dev..

[20] Shaulian E., Karin M. (2002). AP-1 as a regulator of cell life and death. Nat Cell Biol..

[21] Karin M., Liu Z.-G., Zandi E. (1997). AP-1 function and regulation. Curr. Opin. Cell Biol..

[22] Gazon H., Barbeau B., Mesnard J.-M., Peloponese Jr J.-M. (2018). Hijacking of the AP-1 Signaling pathway during development of ATL. Front. Microbiol..

[23] Karin M., Marshall C.J. (1996). The regulation of AP-1 activity by mitogen-activated protein kinases. Phil. Trans. R. Soc. Lond. B.

[24] Tewari D., Nabavi S.F., Nabavi S.M., Sureda A., Farooqi A.A., Atanasov A.G., Vacca R.A., Sethi G., Bishayee A. (2017). Targeting activator protein 1 signaling pathway by bioactive natural agents: Possible therapeutic strategy for cancer prevention and intervention. Pharmacol. Res..

[25] Whitmarsh A.J. (2007). Regulation of gene transcription by mitogen-activated protein kinase signaling pathways. Biochim. Biophys. Acta Mol. Cell Res..

[26] Agron M., Brekhman V., Morgenstern D., Lotan T. (2017). Regulation of AP-1 by MAPK signaling in metal-stressed sea anemone. Cell. Physiol. Biochem..

[27] Drechsler Y., Dolganiuc A., Norkina O., Romics L., Li W., Kodys K., Bach F.H., Mandrekar P., Szabo G. (2006). Heme oxygenase-1 mediates the anti-inflammatory effects of acute alcohol on IL-10 induction involving p38 MAPK activation in monocytes. J. Immunol..

[28] Looby E., Abdel-Latif M.M., Athié-Morales V., Duggan S., Long A., Kelleher D. (2009). Deoxycholate induces COX-2 expression via Erk1/2-, p38-MAPK and AP-1-dependent mechanisms in esophageal cancer cells. BMC Cancer.

[29] Hitchcock L.N., Lattal K.M. (2014). Histone-Mediated Epigenetics in Addiction, Progress in Molecular Biology and Translational Science.

[30] Aletta J.M., Cimato T.R., Ettinger M.J. (1998). Protein methylation: A signal event in post-translational modification. Trends Biochem. Sci..

[31] Elshorbagy A., Jernerén F., Samocha-Bonet D., Refsum H., Heilbronn L. (2016). Serum S-adenosylmethionine, but not methionine, increases in response to overfeeding in humans. Nutr. Diabetes.

[32] Kim J.H., Yoo B.C., Yang W.S., Kim E., Hong S., Cho J.Y. (2016). The role of protein arginine methyltransferases in inflammatory responses. Mediat. Inflamm..

[33] Robertson K.D. (2001). DNA methylation, methyltransferases, and cancer. Oncogene.

[34] Jin B., Li Y., Robertson K.D. (2011). DNA methylation: Superior or subordinate in the epigenetic hierarchy?. Genes Cancer.

[35] Li T., Wang L., Du Y., Xie S., Yang X., Lian F., Zhou Z., Qian C. (2018). Structural and mechanistic insights into UHRF1-mediated DNMT1 activation in the maintenance DNA methylation. Nucleic Acids Res..

[36] Urulangodi M., Mohanty A. (2020). DNA damage response and repair pathway modulation by non-histone protein methylation: Implications in neurodegeneration. J. Cell Commun. Signal..

[37] Lee D.Y., Teyssier C., Strahl B.D., Stallcup M.R. (2005). Role of protein methylation in regulation of transcription. Endocr. Rev..

[38] Hamamoto R., Saloura V., Nakamura Y. (2015). Critical roles of non-histone protein lysine methylation in human tumorigenesis. Nat. Rev. Cancer.

[39] Blanc R.S., Richard S. (2017). Arginine methylation: The coming of age. Mol. Cell.

[40] Barnicle A., Seoighe C., Greally J.M., Golden A., Egan L.J. (2017). Inflammation-associated DNA methylation patterns in epithelium of ulcerative colitis. Epigenetics.

[41] Stresemann C., Lyko F. (2008). Modes of action of the DNA methyltransferase inhibitors azacytidine and decitabine. Int. J. Cancer.

[42] Christman J.K. (2002). 5-Azacytidine and 5-aza-2′-deoxycytidine as inhibitors of DNA methylation: Mechanistic studies and their implications for cancer therapy. Oncogene.

[43] Juttermann R., Li E., Jaenisch R. (1994). Toxicity of 5-aza-2’-deoxycytidine to mammalian cells is mediated primarily by covalent trapping of DNA methyltransferase rather than DNA demethylation. Proc. Natl. Acad. Sci. USA.

[44] Lu R., Wang X., Chen Z.-F., Sun D.-F., Tian X.-Q., Fang J.-Y. (2007). Inhibition of the extracellular signal-regulated kinase/mitogen-activated protein kinase pathway decreases dna methylation in colon cancer cells. J. Biol. Chem..

[45] Pradhan N., Parbin S., Kar S., Das L., Kirtana R., Seshadri G.S., Sengupta D., Deb M., Kausar C., Patra S.K. (2019). Epigenetic silencing of genes enhanced by collective role of reactive oxygen species and MAPK signaling downstream ERK/Snail axis: Ectopic application of hydrogen peroxide repress CDH1 gene by enhanced DNA methyltransferase activity in human breast cancer. Biochim. Biophys. Acta Mol. Basis Dis..

[46] Jin T., Hao J., Fan D. (2018). Nicotine induces aberrant hypermethylation of tumor suppressor genes in pancreatic epithelial ductal cells. Biochem. Biophys. Res. Commun..

[47] Dunn G.P., Rinne M.L., Wykosky J., Genovese G., Quayle S.N., Dunn I.F., Agarwalla P.K., Chheda M.G., Campos B., Wang A. (2012). Emerging insights into the molecular and cellular basis of glioblastoma. Genes Dev..

[48] Heiland D.H., Ferrarese R., Claus R., Dai F., Masilamani A.P., Kling E., Weyerbrock A., Kling T., Nelander S., Carro M.S. (2016). c-Jun-N-terminal phosphorylation regulates DNMT1 expression and genome wide methylation in gliomas. Oncotarget.

[49] Tsai C.-L., Li H.-P., Lu Y.-J., Hsueh C., Liang Y., Chen C.-L., Tsao S.W., Tse K.-P., Yu J.-S., Chang Y.-S. (2006). Activation of DNA Methyltransferase 1 by EBV LMP1 Involves c-Jun NH2-Terminal Kinase Signaling. Cancer Res..

[50] Choudhry P., Mariano M.C., Geng H., Martin T.G., Wolf J.L., Wong S.W., Shah N., Wiita A.P. (2020). DNA methyltransferase inhibitors upregulate CD38 protein expression and enhance daratumumab efficacy in multiple myeloma. Leukemia.

[51] Isakova A., Groux R., Imbeault M., Rainer P., Alpern D., Dainese R., Ambrosini G., Trono D., Bucher P., Deplancke B. (2017). SMiLE-seq identifies binding motifs of single and dimeric transcription factors. Nat. Methods.

[52] Gustems M., Woellmer A., Rothbauer U., Eck S.H., Wieland T., Lutter D., Hammerschmidt W. (2014). c-Jun/c-Fos heterodimers regulate cellular genes via a newly identified class of methylated DNA sequence motifs. Nucleic Acids Res..

[53] Li B., Zhao J., Ma J.-X., Li G.-M., Zhang Y., Xing G.-S., Liu J., Ma X.-L. (2018). Overexpression of DNMT1 leads to hypermethylation of H19 promoter and inhibition of Erk signaling pathway in disuse osteoporosis. Bone.

[54] De La Rica L., Rodríguez-Ubreva J., García M., Islam A.B.M.M.K., Urquiza J.M., Hernando H., Christensen J., Helin K., Gómez-Vaquero C., Ballestar E. (2013). PU.1 target genes undergo Tet2-coupled demethylation and DNMT3b-mediated methylation in monocyte-to-osteoclast differentiation. Genome Biol..

[55] Cai L., Zhan M., Li Q., Li D., Xu Q. (2019). DNA methyltransferase DNMT1 inhibits lipopolysaccharide-induced inflammatory response in human dental pulp cells involving the methylation changes of IL-6 and TRAF6. Mol. Med. Rep..

[56] Meng R., Li D., Feng Z., Xu Q. (2019). MyD88 hypermethylation mediated by DNMT1 is associated with LTA-induced inflammatory response in human odontoblast-like cells. Cell Tissue Res..

[57] Youngblood B., Reich N.O. (2008). The early expressed HIV-1 genes regulate DNMT1 expression. Epigenetics.

[58] Zhou Y., Lu Q. (2008). DNA methylation in T cells from idiopathic lupus and drug-induced lupus patients. Autoimmun. Rev..

[59] Scheinbart L.S., Johnson M.A., Gross L.A., Edelstein S.R., Richardson B.C. (1991). Procainamide inhibits DNA methyltransferase in a human T cell line. J. Rheumatol..

[60] Klinman D.M., Mushinski J.F., Honda M., Ishigatsubo Y., Mountz J.D., Raveche E.S., Steinberg A.D. (1986). Oncogene expression in autoimmune and normal peripheral blood mononuclear cells. J. Exp. Med..

[61] Sunahori K., Nagpal K., Hedrich C.M., Mizui M., Fitzgerald L.M., Tsokos G.C. (2013). The Catalytic subunit of protein phosphatase 2A (PP2Ac) promotes DNA Hypomethylation by suppressing the phosphorylated mitogen-activated protein kinase/Extracellular Signal-regulated Kinase (ERK) Kinase (MEK)/Phosphorylated ERK/DNMT1 Protein pathway in t-cells from controls and systemic lupus erythematosus patients. J. Biol. Chem..

[62] Deng C., Lu Q., Zhang Z., Rao T., Attwood J., Yung R., Richardson B. (2003). Hydralazine may induce autoimmunity by inhibiting extracellular signal-regulated kinase pathway signaling. Arthritis Rheum..

[63] Zhao Q., Wirka R., Nguyen T., Nagao M., Cheng P., Miller C.L., Kim J.B., Pjanic M., Quertermous T. (2019). TCF21 and AP-1 interact through epigenetic modifications to regulate coronary artery disease gene expression. Genome Med..

[64] Da Cunha Jaeger M., Ghisleni E.C., Cardoso P.S., Siniglaglia M., Falcon T., Brunetto A.T., Brunetto A.L., De Farias C.B., Taylor M.D., Nör C. (2020). HDAC and MAPK/ERK Inhibitors cooperate to reduce viability and stemness in medulloblastoma. J. Mol. Neurosci..

[65] Emmons M.F., Faião-Flores F., Sharma R., Thapa R., Messina J.L., Becker J.C., Schadendorf D., Seto E., Sondak V.K., Koomen J.M. (2019). HDAC8 regulates a stress response pathway in melanoma to mediate escape from BRAF inhibitor therapy. Cancer Res..

[66] Louveau B., Jouenne F., De Moura C.R., Sadoux A., Baroudjian B., Delyon J., Herms F., De Masson A., Da Meda L., Battistella M. (2019). Baseline Genomic Features in BRAFV600-mutated metastatic melanoma patients treated with BRAF inhibitor + MEK inhibitor in routine care. Cancers.

[67] Zahreddine H., Borden K.L.B. (2013). Mechanisms and insights into drug resistance in cancer. Front. Pharmacol..

[68] Wang H., Fu C., Du J., Wang H., He R., Yin X., Li H., Li X., Wang H., Li K. (2020). Enhanced histone H3 acetylation of the PD-L1 promoter via the COP1/c-Jun/HDAC3 axis is required for PD-L1 expression in drug-resistant cancer cells. J. Exp. Clin. Cancer Res..

[69] Barski A., Cuddapah S., Cui K., Roh T.-Y., Schones D.E., Wang Z., Wei G., Chepelev I., Zhao K. (2007). High-resolution profiling of histone methylations in the human genome. Cell.

[70] Wagner K.W., Alam H., Dhar S.S., Giri U., Li N., Wei Y., Giri D., Cascone T., Kim J.-H., Ye Y. (2013). KDM2A promotes lung tumorigenesis by epigenetically enhancing ERK1/2 signaling. J. Clin. Investig..

[71] Sun Y., Sun Y., Yue S., Wang Y., Lu F. (2019). Histone deacetylase inhibitors in cancer therapy. Curr. Top. Med. Chem..

[72] Liu W.-J., Chen Y.-J., Chen D.-N., Wu Y.-P., Gao Y.-J., Li J., Zhong W.-J., Jiang L. (2017). A new pair of enantiomeric lignans from the fruits of Morinda citrifolia and their absolute configuration. Nat. Prod. Res..

[73] Iwamoto M., Nakamura Y., Takemura M., Hisaoka-Nakashima K., Morioka N. (2020). TLR4-TAK1-p38 MAPK pathway and HDAC6 regulate the expression of sigma-1 receptors in rat primary cultured microglia. J. Pharmacol. Sci..

[74] Fang W.-F., Chen Y.-M., Lin C.-Y., Huang H.-L., Yeh H., Chang Y.-T., Huang K.-T., Lin M.-C. (2018). Histone deacetylase 2 (HDAC2) attenuates lipopolysaccharide (LPS)-induced inflammation by regulating PAI-1 expression. J. Inflamm..

[75] Tochiki K.K., Cunningham J., Hunt S.P., Géranton S.M. (2012). The expression of spinal methyl-CpG-binding protein 2, DNA Methyltransferases and histone deacetylases is modulated in persistent pain states. Mol. Pain.

[76] Sanna M.D., Galeotti N. (2018). The HDAC1/c-JUN complex is essential in the promotion of nerve injury-induced neuropathic pain through JNK signaling. Eur. J. Pharmacol..

[77] He W., Wu Y., Tang X., Xia Y., He G., Min Z., Li C., Xiong S., Shi Z., Lu Y. (2015). HDAC inhibitors suppress c-Jun/Fra-1-mediated proliferation through transcriptionally downregulating MKK7 and Raf1 in neuroblastoma cells. Oncotarget.

[78] Wu C., Li A., Hu J., Kang J. (2018). Histone deacetylase 2 is essential for LPS-induced inflammatory responses in macrophages. Immunol. Cell Biol..

[79] Harman J.L., Dobnikar L., Chappell J., Stokell B.G., Dalby A., Foote K., Finigan A., Freire-Pritchett P., Taylor A.L., Worssam M.D. (2019). Epigenetic Regulation of vascular smooth muscle cells by Histone H3 Lysine 9 dimethylation attenuates target gene-induction by inflammatory signaling. Arter. Thromb. Vasc. Biol..

[80] De Oliveira S., Boudinot P., Calado Â., Mulero V. (2015). Duox1-Derived H2O2 Modulates Cxcl8 Expression and neutrophil recruitment via JNK/c-JUN/AP-1 signaling and chromatin modifications. J. Immunol..

[81] Renthal W., Nestler E.J. (2008). Epigenetic mechanisms in drug addiction. Trends Mol. Med..

[82] Seidman J.S., Troutman T.D., Sakai M., Gola A., Spann N.J., Bennett H., Bruni C.M., Ouyang Z., Li R.Z., Sun X. (2020). niche-specific reprogramming of epigenetic landscapes drives myeloid cell diversity in nonalcoholic steatohepatitis. Immunity.

[83] Smith E.R., Lee M.G., Winter B., Droz N.M., Eissenberg J.C., Shiekhattar R., Shilatifard A. (2007). Drosophila UTX Is a Histone H3 Lys27 demethylase that colocalizes with the elongating form of RNA polymerase II. Mol. Cell. Biol..

[84] Cheon C.K., Sohn Y.B., Ko J.M., Lee Y.J., Song J.S., Moon J.W., Yang B.K., Ha I.S., Bae E.J., Jin H.-S. (2014). Identification of KMT2D and KDM6A mutations by exome sequencing in Korean patients with Kabuki syndrome. J. Hum. Genet..

[85] Xu B., Mulvey B., Salie M., Yang X., Matsui Y., Nityanandam A., Fan Y., Peng J.C. (2020). UTX/KDM6A suppresses AP-1 and a gliogenesis program during neural differentiation of human pluripotent stem cells. Epigenetics Chromatin.

[86] Hu Z., Huang Y., Liu Y., Sun Y., Zhou Y., Gu M., Chen Y., Xia R., Chen S., Deng A. (2011). β-Arrestin 1 Modulates functions of autoimmune t cells from primary biliary cirrhosis patients. J. Clin. Immunol..

[87] Butler A.A., Johnston D.R., Kaur S., Lubin F.D. (2019). Long noncoding RNA NEAT1 mediates neuronal histone methylation and age-related memory impairment. Sci. Signal..

[88] Shaulian E. (2010). AP-1—The jun proteins: Oncogenes or tumor suppressors in disguise?. Cell. Signal..

[89] Davies C.C., Chakraborty A., Diefenbacher M.E., Skehel M., Behrens A. (2013). Arginine methylation of the c-Jun coactivator RACO-1 is required for c-Jun/AP-1 activation. EMBO J..

[90] Berridge M.J., Bootman M.D., Roderick H.L. (2003). Calcium signalling: Dynamics, homeostasis and remodelling. Nat. Rev. Mol. Cell Biol..

[91] Liu M., Hua W., Chiou Y., Chen C., Yao C., Lai Y., Lin C., Lin W. (2020). Calcium-dependent methylation by PRMT1 promotes erythroid differentiation through the p38α MAPK pathway. FEBS Lett..

[92] Kim E., Jang J., Park J.G., Kim K.-H., Yoon K., Yoo B.C., Cho J.Y. (2020). Protein arginine methyltransferase 1 (PRMT1) Selective Inhibitor, TC-E 5003, has anti-inflammatory properties in TLR4 signaling. Int. J. Mol. Sci..

[93] Hsu J.-M., Chen C.-T., Chou C.-K., Kuo H.-P., Li L.-Y., Lin C.-Y., Lee H.-J., Wang Y.-N., Liu M., Liao H.-W. (2011). Crosstalk between Arg 1175 methylation and Tyr 1173 phosphorylation negatively modulates EGFR-mediated ERK activation. Nat. Cell Biol..

[94] Andreu-Pérez P., Esteve-Puig R., De Torre-Minguela C., López-Fauqued M., Bech-Serra J.J., Tenbaum S., García-Trevijano E.R., Canals F., Merlino G., Ávila M.A. (2011). Protein arginine methyltransferase 5 Regulates ERK1/2 signal transduction amplitude and cell fate through CRAF. Sci. Signal..

[95] Banasavadi-Siddegowda Y.K., Russell L., Frair E., Karkhanis V.A., Relation T., Yoo J.Y., Zhang J., Sif S., Imitola J., Baiocchi R. (2017). PRMT5–PTEN molecular pathway regulates senescence and self-renewal of primary glioblastoma neurosphere cells. Oncogene.

[96] Majumder S., Alinari L., Roy S., Miller T., Datta J., Sif S., Baiocchi R., Jacob S.T. (2009). Methylation of histone H3 and H4 by PRMT5 regulates ribosomal RNA gene transcription. J. Cell. Biochem..

[97] Chatterjee B., Ghosh K., Suresh L., Kanade S.R. (2019). Curcumin ameliorates PRMT5-MEP50 arginine methyltransferase expression by decreasing the Sp1 and NF-YA transcription factors in the A549 and MCF-7 cells. Mol. Cell. Biochem..

[98] Calabretta S., Vogel G., Yu Z., Choquet K., Darbelli L., Nicholson T.B., Kleinman C.L., Richard S. (2018). Loss of PRMT5 promotes PDGFRα degradation during oligodendrocyte differentiation and myelination. Dev. Cell.

[99] Hunter D.J., Schofield D.J., Callander E.J. (2014). The individual and socioeconomic impact of osteoarthritis. Nat. Rev. Rheumatol..

[100] Hunter D.J., Bierma-Zeinstra S. (2019). Osteoarthritis. Lancet.

[101] Dong Y., Wang P., Yang Y., Huang J., Dai Z., Zheng W., Li Z., Yao Z., Zhang H., Zheng J. (2020). PRMT5 inhibition attenuates cartilage degradation by reducing MAPK and NF-κB signaling. Arthritis Res..

[102] Limm K., Ott C., Wallner S., Mueller D.W., Oefner P., Hellerbrand C., Bosserhoff A.-K. (2013). Deregulation of protein methylation in melanoma. Eur. J. Cancer.

[103] Almeida-Rios D., Graça I., Vieira F.Q., Ramalho-Carvalho J., Pereira-Silva E., Martins A.T., Oliveira J., Gonçalves C.S., Costa B.M., Henrique R. (2016). Histone methyltransferase PRMT6 plays an oncogenic role of in prostate cancer. Oncotarget.

[104] Chan L.H., Zhou L., Ng K.Y., Wong T.L., Lee T.K., Sharma R., Loong J.H., Ching Y.P., Yuan Y.-F., Xie D. (2018). PRMT6 Regulates RAS/RAF Binding and MEK/ERK-mediated cancer stemness activities in hepatocellular carcinoma through CRAF methylation. Cell Rep..

